# Characteristics of Populations Included in Randomized Controlled Trials of Hemodiafiltration and Registry Real-Life Populations: A Systematic Review

**DOI:** 10.2215/CJN.0000000855

**Published:** 2025-09-12

**Authors:** Krister Cromm, Ngoc Pham, Tom Yuen, Hanna Jaha, Valeria Saglimbene, Jörgen Hegbrant, Mark Woodward, Andrew Davenport, Bernard Canaud, Claudia Barth, Matthias Rose, Peter J. Blankestijn, Michiel L. Bots, Giovanni F.M. Strippoli

**Affiliations:** 1 Fresenius Medical Care Deutschland GmbH, Global Medical Office, Bad Homburg, Germany; 2 Department of Psychosomatic Medicine, Charité – Universitätsmedizin Berlin, Berlin, Germany; 3 Fresenius Medical Care Asia Pacific Ltd., Global Medical Office, Hong Kong; 4 Department of Precision and Regenerative Medicine and Ionian Area (Dimepre-J), University of Bari, Bari, Italy; 5 School of Public Health, University of Sydney, Sydney, New South Wales, Australia; 6 Division of Nephrology, Department of Clinical Sciences, Lund University, Lund, Sweden; 7 School of Public Health, The George Institute for Global Health, Imperial College London, London, United Kingdom; 8 The George Institute for Global Health, University of New South Wales, Sydney, New South Wales, Australia; 9 Division of Medicine, UCL Centre for Bladder and Kidney Health, Royal Free Hospital, University College London, London, United Kingdom; 10 School of Medicine, Montpellier University, Montpellier, France; 11 Medical Scientific Affairs, B.Braun Avitum, Melsungen, Germany; 12 Department of Nephrology and Hypertension, University Medical Center Utrecht, Utrecht University, Utrecht, The Netherlands; 13 Julius Center for Health Sciences and Primary Care, University Medical Center Utrecht, Utrecht University, Utrecht, The Netherlands

**Keywords:** clinical epidemiology, epidemiology and outcomes, hemodialysis, epidemiologic methods

## Abstract

**Key Points:**

- Coverage of patient characteristics in existing trials of hemodiafiltration versus hemodialysis seems to be broad but incomplete.
- Randomized trials of hemodiafiltration versus hemodialysis have been primarily limited to Europe.
- Inconsistencies between trials and registries support further studies using real-world data to explore applicability of existing evidence.

**Background:**

Selection criteria in randomized trials (randomized controlled trials [RCTs]) can lead to differences in key characteristics between trial participants and real-life populations. We evaluated reporting of population characteristics in existing RCTs of hemodiafiltration (HDF) and in real-life populations included in kidney registries to descriptively identify key differences.

**Methods:**

We used systematic review methodology to identify existing RCTs of HDF versus hemodialysis (1966 to May 2024). We also searched the Fresenius Quantitative Market Analysis team registry database (2024 update) for existing registries from Europe, the Asia-Pacific region, and America including populations on HDF. Patient characteristics from RCTs and registries were extracted, summarized, and compared descriptively.

**Results:**

Eleven RCTs (*N*=5108) and eight registries (*N*=1,147,167) were identified. There were no RCTs in the United States and only two small RCTs from Australia (*N*=124) and Brazil (*N*=195). Most trials were from Europe. Key characteristics consistently reported in both RCTs and registries were only age, sex, diabetes, cardiovascular disease, vascular access type, and dialysis vintage. There was moderate to high heterogeneity for these patient characteristics in RCTs, indicating enrollment of a broad array of people. The proportion of people with diabetes was 26% in RCTs and 43% in registries. The prevalence of arteriovenous fistulas/graft was 90% in RCTs and 70% in registries.

**Conclusions:**

There was a broad but incomplete array of patient characteristics in existing RCTs and real-world registries of HDF versus hemodialysis. Data were primarily limited to Europe and only a core set of demographic and clinical variables. Apart for age, sex, diabetes, cardiovascular disease, vascular access type, and dialysis vintage, other patient and treatment relevant characteristics were erratically or not at all reported in RCTs as well as in real-world registries. With potential differences in patient populations, we support the need for studies examining HDF in real-world settings, *e.g*., with target emulation trials.

## Visual Abstract

**Figure:**
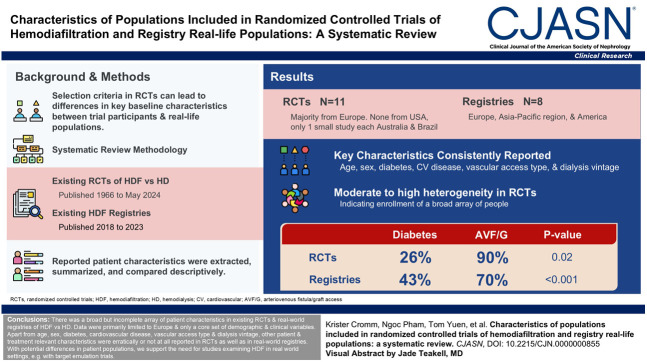

## Introduction

When high-quality evidence from randomized trials becomes available and shows superiority of an intervention compared with existing ones, this may prompt re-evaluation of standards of care. These may be changed to reflect the evidentiary basis and avoid knowledge translation failure.

Many randomized trials are often necessary before policy changes, based on the frequent argument that clinical trials are artificial experiments including highly selected patient populations, with limited generalizability of their findings to real-life scenarios.^1–3^

Several trials are now available on the benefits and harms of hemodiafiltration (HDF) versus HD. The recent “international, multicenter, prospective, randomized, controlled study comparing high-dose HDF versus conventional high-flux hemodialysis (CONVINCE)” trial showed a reduced risk of all-cause death with HDF compared wih high flux HD (hazard ratio, 0.77; 95% confidence interval [CI], 0.65 to 0.93) over a median follow-up of 30 months.^4^ This trial however had a specific inclusion criterion that patients should be good candidates to tolerate high volume HDF (adequate vascular access, ability to tolerate high blood flow rates, *etc.*). The results have been assessed in the context of the totality of evidence and confirms superiority of HDF (all-cause mortality hazard ratio with HDF versus HD 0.84 [95% CI, 0.74 to 0.95]).^5,6^

With health economics and patient priorities data now also released, it is plausible that guidelines may be developed.^7,8^

Given the growing interest in the potential survival and quality-of-life benefits of HDF over conventional HD, there is an urgent need to understand whether randomized trials evaluating HDF adequately reflect the characteristics of real-world populations who may receive this therapy.

In this analysis, we specifically evaluated the reporting of key patient characteristics in existing randomized trials of HDF and in real-world kidney registries. The aim was both to identify if key characteristics of populations were adequately reported in the studies and in the registries as well as if the populations included in randomized controlled trials (RCTs) were only highly selected in comparison with the populations we treat in real life or the characteristics were broadly descriptively comparable.

## Methods

### Inclusion Criteria

We included randomized and quasirandomized (in which allocation to treatment was obtained by alternation, use of alternate medical records, and date of birth or other predictable methods) controlled trials published in any language and comparing HDF versus HD in adults with ESKD where all-cause mortality was a study outcome. We also included real-world kidney registries from Europe, America, and the Asia-Pacific region reporting information in English. Registries focusing only on special populations (*e.g*., only pediatric, only transplantation, only HD, and no HDF) were excluded.

### Search for Randomized Trials

We identified existing randomized trials of HDF versus HD by using search strategies of our Cochrane systematic review of convective versus diffusive treatments for KRT including searches in the Cochrane Central Register of Controlled Trials, the Medical Literature Analysis and Retrieval System Online, and the Excerpta Medica dataBASE (updated to May 2024)^6,9^ (Supplemental Table 1).

### Search for Real-World Kidney Registries from Europe, America, and the Asia-Pacific Region

We identified real-world kidney registries that included prevalent populations on HD and HDF using the 2024 update of the Registries database curated by the Fresenius Quantitative Market Analysis team. Given the absence of a centralized kidney registry database, this was a pragmatic choice for a most comprehensive and systematically updated source (data available on request; Supplemental Table 2). If no data on HDF were available in registries from these geographical regions, we still included the most representative registry from that region.

### Data Screening and Extraction

Two authors (K. Cromm and N. Pham) independently assessed the retrieved titles and abstracts from the searches. The full text (if published) of all potentially relevant studies was retrieved and independently assessed for inclusion. Similarly, two authors (N. Pham and T. Yuen) independently screened the records retrieved from the registry searches. The same authors independently extracted data from identified RCTs and real-world kidney registries using standard data extraction forms. Data on the following baseline patient characteristics were extracted: race and ethnicity (however reported), age (years), sex (male, female), presence of diabetes mellitus (yes, no), presence of cardiovascular disease (yes, no), type of vascular access (arteriovenous fistula [AVF], arteriovenous graft [AVG], central venous catheter), body mass index (BMI; kg/m^2^), and dialysis vintage (month or years). We also extracted, where available, data on the dialysis prescription characteristics including treatment time (min), blood flow rate (ml/min), ultrafiltration volume (L/session), and convective volume (L/session, specifically for HDF).

Discrepancies in Screening and Data Extraction Were Resolved Consulting a Third Experienced Investigator (G.F.M. Strippoli). Authors of RCTs or kidney registry representatives were contacted for key missing data at least twice and for the variables more commonly reported in RCTs and kidney registries. If additional data were obtained, these were included in the analysis.

### Statistical Analysis

For both RCTs and kidney registry datasets, we calculated the mean and 95% CIs for continuous variables (*e.g*., age) and proportions with 95% CIs for categorical variables (*e.g*., sex, diabetes, cardiovascular disease, and vascular access type). All summary statistics were reported separately for the RCT and registry groups.

To visually compare study and registry data, we constructed forest plots displaying individual study or registry estimates with their corresponding 95% CIs. For pooled estimates, we applied an inverse-variance weighting method, which assigns more weight to estimates with greater precision (*i.e*., lower variance). This method is standard in meta-analyses and ensures that larger and more precise studies contribute proportionally more to the overall summary effect.^10^

To quantify heterogeneity across studies and registries,^11,12^ we calculated the I^2^ statistic, which describes the proportion of total variation across studies due to heterogeneity rather than chance. I^2^ values of approximately 25%, 50%, and 75% are interpreted as low, moderate, and high heterogeneity, respectively. We also report the Cochrane Q test, a chi-squared test used to assess whether observed differences across groups are compatible with chance alone. A *P* value < 0.05^13^ in the Q test was considered statistically significant for heterogeneity.

To assess statistical differences in patient characteristics between the RCT and registry groups, we conducted subgroup Q-tests for each variable. This omnibus test compares the between-group variance (RCTs versus registries) relative to the within-group variance and is a standard method for comparing subgroups in meta-analysis.

Where the included studies and registries used different measurement scales and population distributions and to ensure scale-invariant comparison across studies, we additionally calculated standardized mean differences where data permitted. Standardized mean differences are useful for comparing effect sizes across studies with different units of measurement and were computed using Cohen's d or Cohen's h method.^14^ All statistical analyses were conducted using R (version 4.3.0) and MedCalc Statistical Software (version 22.023). Plots were generated using the meta and metafor packages in R.^15^

## Results

### Searching for RCTs and Registries

**Figure 1:**
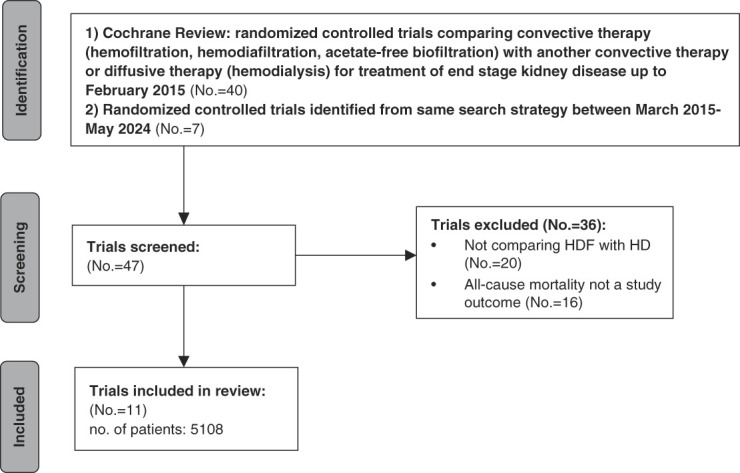
**Flow diagram of the search strategy and result for RCTs.** HD, hemodialysis; HDF, hemodiafiltration; RCT, randomized controlled trial.

The results of our literature search for the identification of RCTs of HDF versus HD are presented in Figure 1. We screened a total of 47 RCTs published up to May 2024, and 20 were excluded as they did not compare HDF with HD and 16 were excluded because all-cause mortality was not a study outcome. The remaining 11 RCTs^4,16–25^ were eligible for analysis. These studies were published between 1994 and 2023 and included a total of 5108 participants (ranging between 44 and 1360 per individual study) from 14 countries, 10 European (France, Germany, Hungary, Italy, The Netherlands, Portugal, Romania, Spain, Turkey, and the United Kingdom) and 4 non-European (Australia, Brazil, Canada, and Norway). Authors of eight studies^16–18,20,21,23–25^ were contacted for missing data with five responding. Two provided additional data^23,25^ and three indicated that data were not retrievable.^16,18,21^

**Figure 2:**
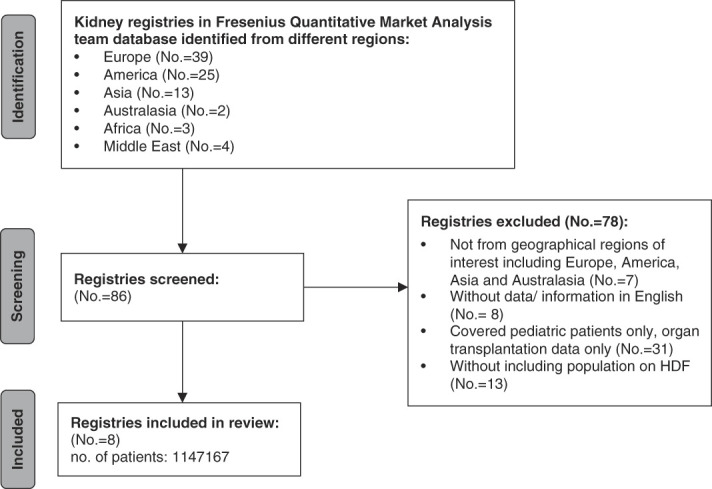
**Flow diagram of the search strategy and result for kidney registries.** Although USRDS registry did not include a HDF population, it is the only key registry within America; hence, it has not been excluded from the analysis. USRDS, United States Renal Data System.

The search for identification of real-world kidney registries in the Fresenius Medical Care Quantitative Market Analysis team database identified a total of 86 registries (Figure 2). Of these, seven were excluded as they were outside of Europe, America, and the Asia-Pacific region; eight due to language restriction criteria (English only); 31 as they covered pediatric patients or organ transplantation data only; and 13 as they reported no data on HDF. As a result, eight registries^26–33^ were eligible for analysis. Given that there were no data on HDF in kidney registries from America, we included the United States Renal Data System (USRDS) registry to provide some indication of the characteristics of populations within this geographical area.

The registries' annual reports covered data from 1,147,167 patients, collected in the years 2018–2022. On our contact with the registry representatives, four^30–33^ provided all missing data for baseline patient characteristics of patients included in the registries: two^26,28^ replied with partial data retrievable or data that were not collected by the registry, one replied that data were not retrievable,^29^ and one^27^ did not respond.

### Randomized Trials of HDF versus HD; Populations and Dialysis Prescription Characteristics

The characteristics of patients enrolled in the 11 trials of HDF versus HD and baseline dialysis prescription features are presented in Table 1 and Supplemental Table 3. Most RCTs were undertaken in Europe, and there were none from the United States.

**Table 1:** Baseline characteristics of people enrolled in randomized controlled trials of hemodiafiltration versus hemodialysis

Study ID^a^	Region	No. of Patients	Age (Mean Years±SD)	Sex—Female, *n* (%)	Cardiovascular Diseases, *n* (%)^b^	Diabetes Mellitus, *n* (%)	AVF or AVG as Vascular Access, *n* (%)	Dialysis Vintage (Mean Years±SD)
Locatelli *et al.*, 1994^16^	Italy	380	53.2±12.9	131 (35)	Not available	20 (5)	Not available	Not available
Wizemann *et al.*, 2000^17^	Germany	44	60.5±11.5	44 (43)	Not available	8 (18)	Not available	Not available
Bolasco 2003^18^	Italy	146	63.9±11.9	62 (43)	Not available	26 (18)	Not available	4.0±4.7^c^
CONTRAST (Dutch) study 2005^19^	The Netherlands^d^, Canada^d^, and Norway^d^	714	64.1±13.7	269 (38)	313 (44)	170 (24)	667 (93)	2.9±2.9
Schiffl 2007^20^	Germany	76	62.0±10.0	34 (45)	Not available	Not available	Not available	2.2±3.8^e^
ESHOL study 2011^21^	Spain	906	65.4±14.4	300 (33)	Not available	226 (25)	813 (90)	2.7±2.9^c^
TURKISH HDF 2013^22^	Turkey	782	56.5±13.9	321 (41)	206 (26)	271 (35)	Not available	4.8±3.7
FRENCHIE 2017^23^	France	381	76.2±6.4	152 (40)	253 (66)	147 (39)	Not available	4.8±5.5
FINESSE 2019^24^	Australia	124	63.5±14.7	55 (44)	Not available	44 (36)	108 (87)	3.4±3.0^f^
HDFIT 2019^25^	Brazil	195	53.0±15.1	56 (29)	43 (22)	68 (35)	Not available	Not available
CONVINCE 2023^4^	Europe^g^ and the United Kingdom	1360	62.4±13.5	504 (36)	612 (45)	481 (35)	1176 (87)	3.3±3.5^c^

AVF, arteriovenous fistula; AVG, arteriovenous graft; CONVINCE, international, multicenter, prospective, randomized, controlled study comparing high-dose HDF versus conventional high-flux hemodialysis; CONTRAST, Dutch CONvective TRAnsport Study; ESHOL, Estudio de Supervivencia de Hemodiafiltración On-Line; FINESSE, Filtration In the Neuropathy of End-Stage kidney disease Symptom Evolution; FRENCHIE, French Convective versus Hemodialysis in Elderly; HDF, hemodiafiltration; HDFIT, Impact of HemoDiaFIlTration on Physical Activity and Self-Reported Outcomes.

a Naming of study IDs is based on the one used in Cochrane review on hemodiafiltration, hemofiltration, and hemodialysis for ESKD (2015).^9^

b Cardiovascular diseases include history of any one or more of the following: ischemic heart diseases (ischemic cardiopathy), congestive heart failure, arrythmias, cerebrovascular diseases, peripheral arterial diseases/peripheral vascular diseases, kidney artery diseases, and valvular heart diseases.

c Assuming dialysis vintage follows a normal distribution, the median (interquartile range) extracted from the randomized controlled trials was converted into mean±SD.^34,35^

d The Netherlands (26 clinics), Canada (two clinics), and Norway (one clinic).

e Assuming dialysis vintage follows a normal distribution, the median (range) extracted from the randomized controlled trial was converted into mean±SD.^34,36^

f Assuming dialysis vintage follows a normal distribution, the median (range) extracted from each study group (hemodialysis or hemodiafiltration) of the randomized controlled trial was converted into mean±SD. Then, it was combined into an overall mean±SD.^34,36^

g Including France, Germany, Hungary, The Netherlands, Spain, Portugal, and Romania.

Detailed information on race and ethnicity was only available in the CONVINCE, the “Dutch CONvective TRAnsport Study”, “Filtration In the Neuropathy of End-Stage kidney disease Symptom Evolution” (FINESSE), and “Impact of HDF on Physical Activity and Self-Reported Outcomes” multicenter RCTs. The remaining trials either enrolled patients from a single country (*e.g*., France, Turkey, and Germany) or did not report race and ethnicity (Supplemental Table 3). Overall, there was underrepresentation of non-White populations in RCTs.

The mean age of patients included in the RCTs ranged between 53 and 76 years. The “French Convective versus Hemodialysis in Elderly” (FRENCHIE) RCT^23^ included an older population (mean age 76 years) than others due to its specific inclusion criteria of people with 65 years or older. There was high coverage of age categories within the identified RCTs, as indicated by the existence of very high heterogeneity between the trials for age (I^2^=99.5%, *P* < 0.001).

The proportion of women in the RCTs was relatively consistent ranging from 29% to 45% with moderate to high heterogeneity between RCTs (I^2^=63.4%, *P* = 0.002).

A highly heterogeneous distribution was also noticed in the prevalence of cardiovascular disease, ranging from 22% to 66% (I^2^=98.4%, *P* < 0.001). Patients reporting higher prevalence of cardiovascular disease (66%) were enrolled in the FRENCHIE (2017) trial, likely due to the higher age (65 years or older) set in the inclusion criteria for this trial. On the contrary, there was a smaller proportion (26%) of people with baseline cardiovascular disease recruited in the Mortality and cardiovascular events in online haemodiafiltration (OL-HDF) compared with high-flux dialysis: results from the Turkish OL-HDF Study (2013)^22^ trial, which included a younger (mean age 57 years) population.

The distribution of patients with diabetes mellitus across the RCTs was also highly heterogeneous (I^2^=96.5%, *P* < 0.001), with RCTs recruiting 5%–39% of diabetic patients.

There was moderate to high heterogeneity in the distribution of people dialyzing with an AVF or AVG for vascular access, with proportions being relatively high and ranging between 87% and 93% (I^2^=88.6%, *P* < 0.001). Data were missing for 7 of 11 RCTs.

Dialysis vintage was also reported in most trials and registries. Across RCTs, dialysis vintage ranged from 2.2 to 4.8 years, with high between-study variability (I^2^=97.3%, *P* < 0.001). The FRENCHIE (2017) trial^23^ and Mortality and cardiovascular events in online haemodiafiltration (OL-HDF) compared with high-flux dialysis: results from the Turkish OL-HDF Study (2013)^22^ trial both reported the longest mean vintage (4.8 years), whereas most other RCTs (*e.g*., Bolasco [2003],^18^ FINESSE [2019],^24^ CONVINCE^4^) clustered around 3–4 years. By contrast, registry studies consistently showed a shorter mean vintage, between 2.9 and 7.4 years.

BMI was reported in five trials,^4,19,22-24^ ranging from 24.8 to 27.4 kg/m^2^, suggesting limited variability.

Regarding dialysis prescription, parameters were variably reported. Treatment time was reported in seven trials,^4,17–19,21,23,25^ ranging between 227 and 273 minutes, indicating general consistency. Blood flow rates were reported in nine trials with inclusion criteria ranging from 250 ml/min to 500 ml/min, typically centered around 300–400 ml/min. Ultrafiltration volume was reported in two trials, ranging from 2.8 to 3.2 L/session, with little variability. Convective volume was reported in only six trials, with considerable differences: high convective volume (23 L/session or higher) was achieved in the “On-Line HDF Survival Study or Estudio de Supervivencia de Hemodiafiltración On-Line (ESHOL)” (23.9 L),^21^ FINESSE (24.7 L),^24^ CONVINCE (25.2 L),^4^ and Impact of HemoDiaFIlTration on Physical Activity and Self-Reported Outcomes (HDFIT) (27.5 L)^25^ trials. For several other trials, convective volumes for HDF were not reported.^16–18,20,22^ Trials using higher convection volumes tended to include younger patients and had slightly lower cardiovascular comorbidity prevalence, although these differences were not statistically significant. The most pronounced difference was observed in vascular access, with higher fistula use in high-volume HDF trials (*P* = 0.001; Supplemental Table 4).

### Real-World Kidney Registries from Europe, America, and the Asia-Pacific Region

The characteristics of people included in the registries are presented in Table 2 and Supplemental Table 5.

**Table 2:** Baseline characteristics of people from major kidney registries annual reports

Registry ID	Region	No. of Patients	Age (Mean Years±SD)	Sex—Female, *n* (%)	Cardiovascular Diseases, *n* (%)	Diabetes Mellitus, *n* (%)	AVF or AVG as Vascular Access, *n* (%)^a^	Dialysis Vintage (Mean Years±SD)
USRDS^b^	America	475,142	62.9^c^	196,845 (41)	367,284 (77)	Not available^d^	365,384 (77)	2.9
JRDR^e^	Asia	316,113	68.9±12.4	109,241 (35)	Not available	155,846 (49)	Not available	7.4
ERA registry^f^	Europe	310,686	67.1±15.2	118,060 (38)	Not available	68,350 (22)	Not available	4.7±4.9
UKRR^g^	Europe	26,405	Not available^h^	9996 (38)	Not available	Not available	Not available	Not available
ANZDATA—Australia^i^	Australasia	12,861	63.6±14.8	5088 (40)	7181 (56)	7077 (55)	10,288 (80)	1.6±3.1
ANZDATA—New Zealand^j^	Australasia	2389	58.6±14.3	980 (41)	1205 (51)	1448 (61)	1313 (55)	2.1±3.6
SRR^k^	Europe	1937	63.4±14.5	757 (39)	960 (50)	776 (40)	1055 (55)	3.7±3.9
FIRR^l^	Europe	1634	66.2±14.8	572 (36)	582 (39)	525 (32)	1313 (80)	5.2±6.6

ANZDATA, Australia and New Zealand Dialysis and Transplant Registry; AVF, arteriovenous fistula; AVG, arteriovenous graft; ERA, European Renal Association; FIRR, Finnish Registry for Kidney Diseases; JRDR, Japanese Society for Dialysis Therapy Renal Data Registry; SRR, Scottish Renal Registry; UKRR, UK Renal Registry; USRDS, United States Renal Data System.

a For many registries, only combined data of arteriovenous fistula and arteriovenous graft were available.

b 2023 annual data report—data updated to December 31, 2021, patients on hemodiafiltration were not included as no data available in the United States.

c SD not available.

d Data were only available for the overall population on incident patients with ESKD and people with stage G3–G4 CKD.

e 2018 annual dialysis data report—data updated to December 31, 2018.

f 2021 Annual report—data updated to December 31, 2021.

g 25th annual report—data updated to December 31, 2021.

h Only median age available (66.0).

i 46th annual report 2023—data updated to December 31, 2022.

j 46th annual report 2023—data updated to December 31, 2022.

k 2023 Annual report—data updated to December 31, 2022.

l 2021 Annual report—data updated to December 31, 2021.

Detailed information about race and ethnicity was only available in the UK Renal Registry and the USRDS. The UK Renal Registry reported a patient distribution of 69% White, 15% Asian, 12% Black, and 4% other ethnicities. The USRDS reported 37% White, 5% Asian, 34% Black, and 24% other ethnicities. Data from other registries were sparse or incomplete and are presented in Supplemental Table 5.

The mean age ranged from 59 to 69 years (I^2^=99.9%, *P* < 0.001) with data missing for 2 of 8 registries. The proportion of women included ranged from 35% to 41% (I^2^=99.8%, *P* < 0.001). The prevalence of patients with cardiovascular disease and diabetes mellitus comorbidities ranged from 39% to 77% (I^2^=99.9%, *P* < 0.001) and 22% to 61% (I^2^=99.9%, *P* < 0.001), respectively. Data for cardiovascular disease were missing for three registries, and data for diabetes were missing for two registries.

The type of vascular access used was assessed by the prevalence of patients using AVF or AVG because only combined data were available. The relative weight of each registry's contribution varied with individual estimates ranging from 55% to 80%. The analysis demonstrated significant heterogeneity (I^2^=99.6%, *P* < 0.001). Vascular access data were missing for three registries.

Dialysis vintage was available for seven registries.^26–28,30–33^ The mean dialysis vintage ranged from 1.6 to 7.4 years, with the “Australia and New Zealand Dialysis and Transplant Registry (ANZDATA)” Australia registry reporting the shortest average duration (1.64 years) and the Japanese Registry the longest (7.4 years). The UK registry did not report dialysis vintage. As with other characteristics, dialysis vintage varied significantly across registries (I^2^=99.5%, *P*<0.001).

BMI was only sparsely reported. Among the registries that where checked, no BMI data were available for detailed analysis. Therefore, no heterogeneity analysis was conducted for BMI.

### Descriptive Comparison of the Characteristics of Populations Included in RCTs and Those Included in Real-World Kidney Registries

**Figure 3:**
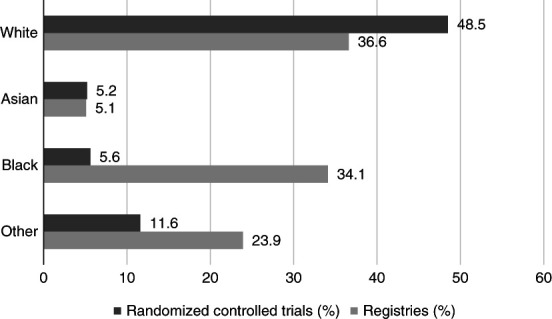
Disparities in race and ethnicity representation between RCTs and registries in kidney disease populations (%).

Although race and ethnicity was reported in only three RCTs and two registries, a descriptive analysis of existing data shows a racial disparity in clinical trial enrollment, with White participants overrepresented and Black participants and other racial groups underrepresented in RCTs relative to their real-world proportions in registries (Figure 3).

**Figure 4:**
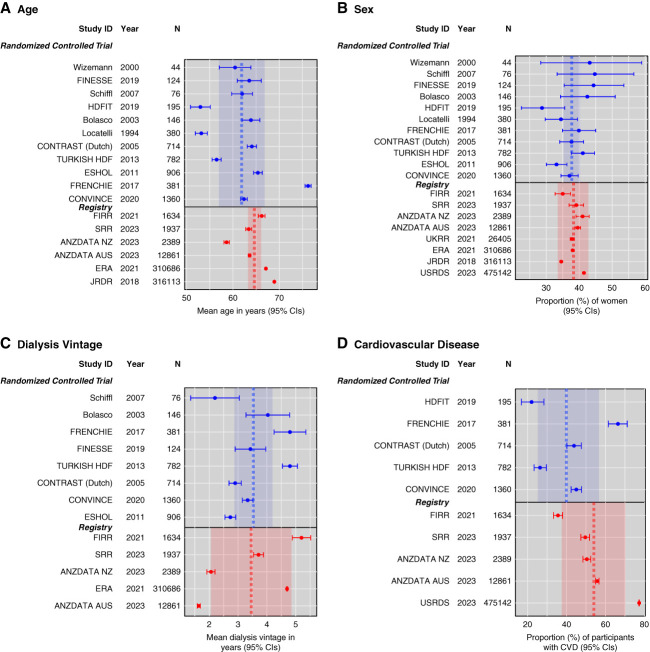

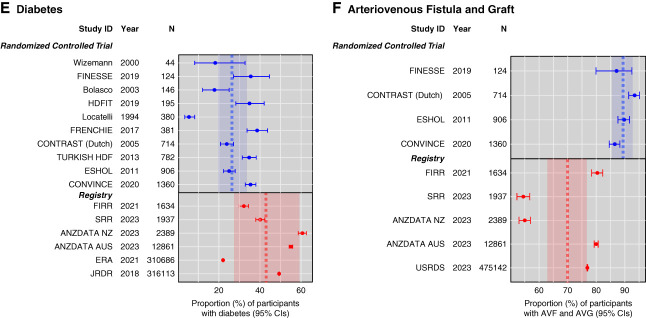
**Visual representation of baseline characteristics of patients included in randomized trials of HDF versus hemodialysis and in kidney registries from Europe, America, and the Asia-Pacific region.** Plots display all individual randomized trials and registry estimates along with their corresponding 95% CIs. Data points in blue belong to the data collected from the randomized trials, while red data points show the values of the registry data. The overall mean values for all characteristics for randomized trials and registries were represented as vertical, dotted lines with the colored boxes in blue, and red indicating the 95% CIs for these overall values. For these values, weights were assigned using the inverse-variance method. In the plots, studies and registries were sorted by their weight, from lowest to highest, to evaluate their contributions to the pooled overall mean. ANZDATA, Australia and New Zealand Dialysis and Transplant Registry; CI, confidence interval; CONVINCE, international, multicenter, prospective, randomized, controlled study comparing high-dose HDF versus conventional high-flux hemodialysis; ESHOL, Estudio de Supervivencia de Hemodiafiltración On-Line; HDFIT, Impact of HemoDiaFIlTration on Physical Activity and Self-Reported Outcomes.

Figure 4 shows forest plots comparing baseline characteristics of populations included in RCTs (blue points) and real-world registries (red points). Each point represents an individual study or registry, with horizontal lines indicating 95% CIs. Data points are ordered by increasing statistical weight, and pooled summary estimates for each group are marked by vertical dashed lines, with shaded areas representing their 95% CIs.

The figure and Table 3 provide a descriptive overview of between-study variability and the degree of overlap between RCT and registry populations. For characteristics such as age and sex, blue and red points are interspersed along the horizontal axis, and the pooled estimates are relatively close. By contrast, for diabetes and vascular access, RCTs and registries show more distinct distributions, with less overlap and more separation between summary lines. Estimates for cardiovascular disease exhibit wide CIs across both study types, reflecting substantial heterogeneity.

**Table 3:** Comparative analyses between estimates of patient baseline characteristics in randomized controlled trials and registries

Characteristic	RCTs Mean or Proportion (95% CI)	Registries Mean or Proportion (95% CI)	SMDs Cohen's *d* or *h* (Registry-RCT) (95% CI)	Difference RCTs versus Registries, *P* Value	Difference RCTs versus Registries (Europe Only), *P* Value
Age	61.9 (58.0 to 65.8)	64.6 (61.7 to 67.6)	0.48 (−0.52 to 1.48)	0.35	0.46
Sex—female	37.8 (35.2 to 40.5)	38.2 (36.4 to 40.0)	0.01 (−0.02 to 0.04)	0.81	0.99
Cardiovascular disease	38.5 (22.8 to 57.1)	54.1 (39.6 to 68.0)	0.31 (0.28 to 0.35)	0.19	0.82
Diabetes mellitus	26.3 (19.8 to 33.4)	42.9 (27.5 to 58.9)	0.35 (0.32 to 0.38)	0.02	0.28
Vascular access (AVF and AVG)	89.6 (85.7 to 92.6)	70.0 (62.9 to 76.6)	−0.50 (0.56 to −0.45)	<0.001	0.03
Dialysis vintage	3.5 (2.9 to 4.2)	3.5 (2.1 to 4.8)	0.00 (−0.03 to 0.03)	0.91	0.13

Cohen's d was used for mean values and Cohen's h for proportions. Both effect sizes are interpreted following standard conventions such as: small (0.2), medium (0.5), and large (0.8). Effect sizes were calculated as the difference between the registry and randomized controlled trial groups (registry minus randomized controlled trial). AVF, arteriovenous fistula; AVG, arteriovenous graft; CI, confidence interval; RCT, randomized controlled trial; SMD, standardized mean difference.

## Discussion

Our review demonstrates that while RCTs of HDF share some similarities with real-world registries, they mainly represent European, lower-risk populations, raising some concerns about the broader generalizability of trial findings to routine clinical practice. We identified 11 RCTs comparing HDF with HD, primarily conducted in Europe, with no RCTs in the United States and only two small RCTs conducted in Australia and Brazil, respectively. We also identified seven kidney registries from Europe and the Asia-Pacific region including real-world data on HDF and no data in the USRDS registry. We descriptively show that there is variability in reported patient characteristics across RCTs of HDF and real-world registries. The study populations within RCTs had overlapping distribution for some (age, sex, and cardiovascular comorbidities) but not all characteristics with registries. The prevalence of diabetes was lower and the proportion of patients using AVF or AVG (as opposed to central venous catheters) was higher in RCTs compared with registries. Overall, these findings indicate that populations included in RCTs may partly but not fully reflect real-world clinical practice, particularly outside of Europe. The lower prevalence of diabetes and higher use of AVF/AVG in RCTs is an indicator that participants included in these trials may be “lower-risk” compared with the populations in real-world settings. The substantially higher proportion of patients with AVFs or AVGs in HDF RCTs compared with real-world registries, likely reflects a technical requirement for achieving high convection volumes (23 L/session or higher) in postdilution HDF. This observation is of relevance given the consistent association between higher convection volumes and greater survival benefit in meta-analyses of HDF trials. These access-related considerations are important when evaluating the applicability of HDF trials to routine clinical practice and should inform patient selection and health system planning for HDF implementation.

Previously, in an individual patient data meta-analysis, we demonstrated that the survival and cardiovascular benefits of HDF were consistent across multiple subgroups including diabetes and type of vascular access.^5^ Nonetheless, the magnitude of benefit can vary.^37^ Therefore, individual patient characteristics should still be considered by clinicians when determining the risk-benefit profile of HDF in real life clinical practice.

Recent trials, including our CONVINCE study, have found that HDF offers advantages beyond survival, including improved health-related quality of life.^4,5,7^ These findings support the case for increasing adoption of HDF. As noted, CONVINCE and RCTs in general apply specific inclusion criteria and may therefore represent a subset of patients who are fitter or more adherent to treatment, which limits generalizability to broader dialysis populations, particularly those with higher comorbidity burden or catheter dependence. Our analysis has identified also significant gaps in the evidentiary basis in non-European regions (*e.g*., the United States and the Asia-Pacific region, where direct RCT evidence is lacking or suboptimal at best). A shift toward broader implementation would require additional research, particularly in the lines of economic evaluations.

Health economic considerations relating to the CONVINCE RCT have also recently been released. The trial was conducted mainly in a European setting, limiting generalizability.^8^ Similarly, although a comprehensive report on patient-reported outcomes of the CONVINCE trial has been released, this is largely restricted to the CONVINCE RCT itself.^7^ An additional RCT of HDF versus HD is ongoing in the United Kingdom.^38^ The likelihood of further large-scale RCTs in other countries such as the United States is low, due to many central barriers facing the national clinical research enterprise.^39^

The observed differences found in our study in any case reinforce the need for additional evidence, perhaps in the form of target trial emulation, and virtual trials that examine HDF in real-world settings, particularly in underrepresented regions including the United States and parts of the Asia-Pacific region. This may be a more practical approach.^40^ These studies can be designed specifically to explore the benefits and harms of HDF in special population subgroups. As RCTs may not capture the full spectrum of clinical complexity encountered in real-world practice, this supports the need for complementary real-world effectiveness studies.

This study systematically synthesized evidence from RCTs and registries using validated methods and rigorous data collection, but its findings are constrained by incomplete reporting, regional imbalances, and limited availability of key clinical variables, which restrict generalizability and depth of comparison.

We reviewed the totality of evidence on HDF versus HD focusing on representativeness of the RCTs for key patient characteristics and similarly with real-world registry data. We used validated methods for systematic reviews and registry identification using a pragmatic but structured approach. Data collection and extraction were conducted independently by two researchers, with discrepancies resolved with a third experienced investigator. We made extensive efforts to retrieve missing data by contacting trial investigators and registry representatives.

However, there were limitations. We excluded HDF RCTs that did not report all-cause mortality in an attempt to focus on hard patient-level end point trials only. This may have limited our ability to capture prevalence data for certain baseline covariates from other RCTs. There were differences in geographic representation between RCTs and registries. Data from all RCTs except two were from Europe while real-world data from kidney registries included populations from Europe (including the United Kingdom) and the Asia-Pacific Region. There were no registry data from the United States. In addition, many variables were not consistently or quantitatively reported across both RCTs and registries, which did not allow formal pooling and permitted only limited comparative statistical testing. We aimed to examine several variables relevant to HDF (*e.g*., blood flow rate, treatment time, substitution volume, frailty measures, or laboratory values), but consistent reporting across RCTs and registries was limited, allowing analysis for only a core set of demographic and clinical variables. This limits generalizability and may underestimate differences in other unmeasured characteristics. We can also not exclude the possibility of bias due to the pooling of data. For merely descriptive reasons, we attempted to visually (forest plots) compare pooled characteristics of the RCTs populations with those of the registries, which may have obscured some regional differences. Restricting analyses to only data from European RCTs and registries resulted in the disappearance of a key difference of diabetes prevalence. Only a difference for type of vascular access use remained, with more patients having AVFs or graft in RCTs compared to registries. While it may be argued that the results of trials are less applicable in settings where fewer patients have fistulas, it should also be argued that programs for increasing prevalence of AVFs are of major importance in real-world settings. We could have also compared RCT and registry data looking at registries reported in non-English language, to achieve a more granular comparison, but this was beyond our scope and possibility in this study. It should be noted that some RCTs and registries may have had missing data, particularly for the characteristics of cardiovascular disease and type of vascular access. We also could only focus on age, sex, cardiovascular disease, diabetic status, type of vascular access and dialysis vintage, the typical subgroup analyses explored in kidney trials, and also the most consistently reported variables. Nonetheless, other factors could have been accounted for, including blood flow rates, time on dialysis (dialysis vintage), residual kidney function, convective volume, and dialysis adequacy. We were unable to do this, due to lack of reporting. Finally, we were only able to collect data on the combined rates of AVF and AVG, despite a potentially different effect on patient outcomes and this affected our ability to report some of the data in our plots.

In conclusion, we show that RCTs of HDF reported a broad array of patient characteristics that partly overlap with those observed in selected real-world registries, particularly in Europe. However, key differences remain, and the populations studied in RCTs may not fully reflect the heterogeneity of real-life dialysis patients.

The evidentiary basis to support the use of HDF over and above high-flux HD is strong and consistent within RCTs. It appears to be applicable to a broad range of patients. As adoption of HDF increases and policies evolve, with a low likelihood that more RCTs will be conducted, real-world effectiveness studies (including target emulation trials), as well as virtual trials will be essential to complement existing data and support equitable implementation across diverse patient populations. Statistical methods including inverse odds of sampling weights, propensity score matching, or transportability frameworks could be applied to quantify how well trial participants reflect broader patient populations. These approaches can estimate population average treatment effects and inform whether reweighting trial samples could yield valid inferences for real-world patients. Access to raw data would also enable subgroup analyses (*e.g*., by age, access type, diabetes status), better adjustment for confounders, and emulation of target trials that are currently unfeasible with aggregate-level data.

## Supplementary Material

## Data Availability

Original data generated for the study will be made available upon reasonable request to the corresponding author. Data Type: Research Protocols; Software Executable Code; Aggregated Data. Reason for Restricted Access: The data that support the findings of this study, the study protocol and statistical codes used in the analysis are available upon request to the corresponding author and will be shared contingent upon methodologic transparency and non-commercial use.

## Supplemental Material

Supplemental Table 1. Search strategy for RCTs of HDF versus HD.

Supplemental Table 2. Characteristics of the 2024 update of the registries database curated by the Fresenius Quantitative Market Analysis team (data/search strategy available on request).

Supplemental Table 3. Descriptive baseline characteristics of people enrolled in RCTs of HDF versus HD.

Supplemental Table 4. Comparative analysis between estimates of patient baseline characteristics in RCTs—sorted by mean convection volume from lowest to highest.

Supplemental Table 5. Descriptive baseline characteristics of people from major kidney registries annual reports.
