# Supplementary material for: Characteristics of Populations Included in Randomized Controlled Trials of Hemodiafiltration and Registry Real-Life Populations: A Systematic Review
Source: Clin J Am Soc Nephrol. 2025 Sep 12;21(1):95–107. doi: 10.2215/CJN.0000000855 (PMC13135059; doi:10.2215/CJN.0000000855)
Supplement: Supplementary file 1 [file cjasn-21-095-s001.pdf]

ASN American Society of Nephrology
JASN Journal of the American Society of Nephrology
KIDNEY360 Accessing Our World From Every Angle
CJASN Clinical Journal of the American Society of Nephrology

ASN Journal Disclosure Form

As per ASN journal policy, I have disclosed any financial relationships or commitments I have held in the past 36 months as included below. I have listed my Current Employer below to indicate there is a relationship requiring disclosure. If no relationship exists, my Current Employer is not listed.

C. Barth reports the following:
Employer: BBraun Avitum AG; Ownership Interest: BBraun SE; Research Funding: BBraun Avitum AG; Advisory or Leadership Role: BBraun Melsungen AG supervisory board; payment received; and Other Interests or Relationships: medical societues: DGfN; ERA; ISN, ASN, EKHA, deutsche Nierenstiftung, Kuratorium DGfN.

I understand that the information above will be published within the journal article, if accepted, and that failure to comply and/or to accurately and completely report the potential financial conflicts of interest could lead to the following: 1) Prior to publication, article rejection, or 2) Post-publication, sanctions ranging from, but not limited to, issuing a correction, reporting the inaccurate information to the authors' institution, banning authors from submitting work to ASN journals for varying lengths of time, and/or retraction of the published work.

Name: Claudia Maria Barth
Manuscript ID: CJASN-2025-000425
Manuscript Title: Baseline characteristics of populations included in randomized controlled trials of hemodiafiltration versus registry real-life populations: a systematic review
Date of Completion: June 17, 2025
Disclosure Updated Date: June 17, 2025

ASN
American Society of Nephrology
JASN
Journal of the American Society of Nephrology
KIDNEY360
Accessing Our World From Every Angle
CJASN
Clinical Journal of the American Society of Nephrology

ASN Journal Disclosure Form

As per ASN journal policy, I have disclosed any financial relationships or commitments I have held in the past 36 months as included below. I have listed my Current Employer below to indicate there is a relationship requiring disclosure. If no relationship exists, my Current Employer is not listed.

P. Blankestijn reports the following:
Consultancy: Medtronic, Fresenius, MSD; Honoraria: Medtronic, Fresenius, MSD; Advisory or Leadership Role: MSD; and Speakers Bureau: Fresenius.

I understand that the information above will be published within the journal article, if accepted, and that failure to comply and/or to accurately and completely report the potential financial conflicts of interest could lead to the following: 1) Prior to publication, article rejection, or 2) Post-publication, sanctions ranging from, but not limited to, issuing a correction, reporting the inaccurate information to the authors' institution, banning authors from submitting work to ASN journals for varying lengths of time, and/or retraction of the published work.

Name: Peter J. Blankestijn
Manuscript ID: CJASN-2025-000425R1
Manuscript Title: Characteristics of populations included in randomized controlled trials of hemodiafiltration and registry real-life populations: a systematic review,
Date of Completion: August 6, 2025
Disclosure Updated Date: May 25, 2025

ASN
American Society of Nephrology
JASN
Journal of the American Society of Nephrology
KIDNEY360
Accessing Our World From Every Angle
CJASN
Clinical Journal of the American Society of Nephrology

ASN Journal Disclosure Form

As per ASN journal policy, I have disclosed any financial relationships or commitments I have held in the past 36 months as included below. I have listed my Current Employer below to indicate there is a relationship requiring disclosure. If no relationship exists, my Current Employer is not listed.

M. Bots reports the following:
Employer: University Medical Center Utrecht

I understand that the information above will be published within the journal article, if accepted, and that failure to comply and/or to accurately and completely report the potential financial conflicts of interest could lead to the following: 1) Prior to publication, article rejection, or 2) Post-publication, sanctions ranging from, but not limited to, issuing a correction, reporting the inaccurate information to the authors' institution, banning authors from submitting work to ASN journals for varying lengths of time, and/or retraction of the published work.

Name: Michiel L. Bots
Manuscript ID: CJASN-2025-000425R2
Manuscript Title: Characteristics of populations included in randomized controlled trials of hemodiafiltration and registry real-life populations: a systematic review
Date of Completion: August 26, 2025
Disclosure Updated Date: August 26, 2025

ASN
American Society of Nephrology
JASN
Journal of the American Society of Nephrology
KIDNEY360
Accessing Our World From Every Angle
CJASN
Clinical Journal of the American Society of Nephrology

ASN Journal Disclosure Form

As per ASN journal policy, I have disclosed any financial relationships or commitments I have held in the past 36 months as included below. I have listed my Current Employer below to indicate there is a relationship requiring disclosure. If no relationship exists, my Current Employer is not listed.

B. Canaud reports the following:
Employer: Scientific consultant for Fresenius Medical Care, Germany up to December 2022 - Retired from December 2022; Consultancy: Senior scientist consultant for Fresenius Medical Care up to December 2022; and Other Interests or Relationships: CEO of MTX Consulting Int, Montpellier-France.

I understand that the information above will be published within the journal article, if accepted, and that failure to comply and/or to accurately and completely report the potential financial conflicts of interest could lead to the following: 1) Prior to publication, article rejection, or 2) Post-publication, sanctions ranging from, but not limited to, issuing a correction, reporting the inaccurate information to the authors' institution, banning authors from submitting work to ASN journals for varying lengths of time, and/or retraction of the published work.

Name: Bernard J. Canaud
Manuscript ID: CJASN-2025-000425R2
Manuscript Title: Characteristics of populations included in randomized controlled trials of hemodiafiltration and registry real-life populations: a systematic review.
Date of Completion: August 25, 2025
Disclosure Updated Date: July 8, 2025

ASN American Society of Nephrology | JASN Journal of the American Society of Nephrology | KIDNEY360 Accessing Our World From Every Angle | CJASN Clinical Journal of the American Society of Nephrology

ASN Journal Disclosure Form

As per ASN journal policy, I have disclosed any financial relationships or commitments I have held in the past 36 months as included below. I have listed my Current Employer below to indicate there is a relationship requiring disclosure. If no relationship exists, my Current Employer is not listed.

K. Cromm reports the following:
Employer: Fresenius Medical Care; Ownership Interest: Fresenius Medical Care; and Research Funding: Fresenius Medical Care.

I understand that the information above will be published within the journal article, if accepted, and that failure to comply and/or to accurately and completely report the potential financial conflicts of interest could lead to the following: 1) Prior to publication, article rejection, or 2) Post-publication, sanctions ranging from, but not limited to, issuing a correction, reporting the inaccurate information to the authors' institution, banning authors from submitting work to ASN journals for varying lengths of time, and/or retraction of the published work.

Name: Krister Cromm
Manuscript ID: CJASN-2025-000425
Manuscript Title: Baseline characteristics of populations included in randomized controlled trials of hemodiafiltration versus registry real-life populations: a systematic review
Date of Completion: June 17, 2025
Disclosure Updated Date: June 17, 2025

ASN American Society of Nephrology | JASN Journal of the American Society of Nephrology | KIDNEY360 Accessing Our World From Every Angle | CJASN Clinical Journal of the American Society of Nephrology

ASN Journal Disclosure Form

As per ASN journal policy, I have disclosed any financial relationships or commitments I have held in the past 36 months as included below. I have listed my Current Employer below to indicate there is a relationship requiring disclosure. If no relationship exists, my Current Employer is not listed.

A. Davenport reports the following:
Consultancy: 1. Fresenius Medical Care - lecture on haemodiafiltration; 2. Nipro Corporation - lecture on alpha 1 macroglobulin; Ownership Interest: United utilities Ltd shares; Astra Zenica shares; Honoraria: 1. Fresenius Medical Company; 2. Nipro Corporation; and Advisory or Leadership Role: Advisory board -WAK Scientific advisory board; Leadership positions; European Dialysis & Transplant Association ERN committee ; past president - International Society for Hemodialysis,.

I understand that the information above will be published within the journal article, if accepted, and that failure to comply and/or to accurately and completely report the potential financial conflicts of interest could lead to the following: 1) Prior to publication, article rejection, or 2) Post-publication, sanctions ranging from, but not limited to, issuing a correction, reporting the inaccurate information to the authors' institution, banning authors from submitting work to ASN journals for varying lengths of time, and/or retraction of the published work.

Name: Andrew Davenport
Manuscript ID: CJASN-2025-000425
Manuscript Title: Baseline characteristics of populations included in randomized controlled trials of hemodiafiltration versus registry real-life populations: a systematic review
Date of Completion: June 17, 2025
Disclosure Updated Date: June 17, 2025

ASN American Society of Nephrology | JASN Journal of the American Society of Nephrology | KIDNEY360 Accessing Our World From Every Angle | CJASN Clinical Journal of the American Society of Nephrology

ASN Journal Disclosure Form

As per ASN journal policy, I have disclosed any financial relationships or commitments I have held in the past 36 months as included below. I have listed my Current Employer below to indicate there is a relationship requiring disclosure. If no relationship exists, my Current Employer is not listed.

J. Hegbrant reports the following:
Employer: JBA Medical AB; Consultancy: Triomed AB; Ownership Interest: Redsense Medical AB ; Triomed AB ; LundaTec AB ; NorrDia AB; and Advisory or Leadership Role: NorrDia AB; Triomed AB.

I understand that the information above will be published within the journal article, if accepted, and that failure to comply and/or to accurately and completely report the potential financial conflicts of interest could lead to the following: 1) Prior to publication, article rejection, or 2) Post-publication, sanctions ranging from, but not limited to, issuing a correction, reporting the inaccurate information to the authors' institution, banning authors from submitting work to ASN journals for varying lengths of time, and/or retraction of the published work.

Name: Jorgen BA Hegbrant
Manuscript ID: CJASN-2025-000425R1
Manuscript Title: Characteristics of populations included in randomized controlled trials of hemodiafiltration and registry real-life populations: a systematic review,
Date of Completion: August 19, 2025
Disclosure Updated Date: June 18, 2025

ASN American Society of Nephrology
JASN Journal of the American Society of Nephrology
KIDNEY360 Accessing Our World From Every Angle
CJASN Clinical Journal of the American Society of Nephrology

ASN Journal Disclosure Form

As per ASN journal policy, I have disclosed any financial relationships or commitments I have held in the past 36 months as included below. I have listed my Current Employer below to indicate there is a relationship requiring disclosure. If no relationship exists, my Current Employer is not listed.

H. Jaha reports the following:
Employer: Fresenius Medical Care

I understand that the information above will be published within the journal article, if accepted, and that failure to comply and/or to accurately and completely report the potential financial conflicts of interest could lead to the following: 1) Prior to publication, article rejection, or 2) Post-publication, sanctions ranging from, but not limited to, issuing a correction, reporting the inaccurate information to the authors' institution, banning authors from submitting work to ASN journals for varying lengths of time, and/or retraction of the published work.

Name: Hanna Jaha
Manuscript ID: CJASN-2025-000425R2
Manuscript Title: Characteristics of populations included in randomized controlled trials of hemodiafiltration and registry real-life populations: a systematic review.
Date of Completion: August 22, 2025
Disclosure Updated Date: June 25, 2025

ASN American Society of Nephrology | JASN Journal of the American Society of Nephrology | KIDNEY360 Accessing Our World From Every Angle | CJASN Clinical Journal of the American Society of Nephrology

ASN Journal Disclosure Form

As per ASN journal policy, I have disclosed any financial relationships or commitments I have held in the past 36 months as included below. I have listed my Current Employer below to indicate there is a relationship requiring disclosure. If no relationship exists, my Current Employer is not listed.

N. Pham reports the following:
Employer: Fresenius Medical Care; and Research Funding: Fresenius Medical Care.

I understand that the information above will be published within the journal article, if accepted, and that failure to comply and/or to accurately and completely report the potential financial conflicts of interest could lead to the following: 1) Prior to publication, article rejection, or 2) Post-publication, sanctions ranging from, but not limited to, issuing a correction, reporting the inaccurate information to the authors' institution, banning authors from submitting work to ASN journals for varying lengths of time, and/or retraction of the published work.

Name: Ngoc Pham
Manuscript ID: CJASN-2025-000425R1
Manuscript Title: Characteristics of populations included in randomized controlled trials of hemodiafiltration and registry real-life populations: a systematic review
Date of Completion: August 4, 2025
Disclosure Updated Date: September 6, 2024

ASN American Society of Nephrology JASN Journal of the American Society of Nephrology KIDNEY360 Accessing Our World From Every Angle CJASN Clinical Journal of the American Society of Nephrology

ASN Journal Disclosure Form

As per ASN journal policy, I have disclosed any financial relationships or commitments I have held in the past 36 months as included below. I have listed my Current Employer below to indicate there is a relationship requiring disclosure. If no relationship exists, my Current Employer is not listed.

M. Rose reports the following:
Employer: Charité - Universitätsmedizin Berlin; Consultancy: Astra Zeneca; and Honoraria: Novo Nordisk.

I understand that the information above will be published within the journal article, if accepted, and that failure to comply and/or to accurately and completely report the potential financial conflicts of interest could lead to the following: 1) Prior to publication, article rejection, or 2) Post-publication, sanctions ranging from, but not limited to, issuing a correction, reporting the inaccurate information to the authors' institution, banning authors from submitting work to ASN journals for varying lengths of time, and/or retraction of the published work.

Name: Matthias Rose
Manuscript ID: CJASN-2025-000425
Manuscript Title: Baseline characteristics of populations included in randomized controlled trials of hemodiafiltration versus registry real-life populations: a systematic review
Date of Completion: June 18, 2025
Disclosure Updated Date: June 18, 2025

ASN American Society of Nephrology | JASN Journal of the American Society of Nephrology | KIDNEY360 Accessing Our World From Every Angle | CJASN Clinical Journal of the American Society of Nephrology

ASN Journal Disclosure Form

As per ASN journal policy, I have disclosed any financial relationships or commitments I have held in the past 36 months as included below. I have listed my Current Employer below to indicate there is a relationship requiring disclosure. If no relationship exists, my Current Employer is not listed.

V. Saglimbene reports the following:
Employer: IQVIA Solution

I understand that the information above will be published within the journal article, if accepted, and that failure to comply and/or to accurately and completely report the potential financial conflicts of interest could lead to the following: 1) Prior to publication, article rejection, or 2) Post-publication, sanctions ranging from, but not limited to, issuing a correction, reporting the inaccurate information to the authors' institution, banning authors from submitting work to ASN journals for varying lengths of time, and/or retraction of the published work.

Name: Valeria Maria Saglimbene
Manuscript ID: CJASN-2025-000425R2
Manuscript Title: Characteristics of populations included in randomized controlled trials of hemodiafiltration and registry real-life populations: a systematic review"
Date of Completion: August 29, 2025
Disclosure Updated Date: August 29, 2025

ASN Journal Disclosure Form

As per ASN journal policy, I have disclosed any financial relationships or commitments I have held in the past 36 months as included below. I have listed my Current Employer below to indicate there is a relationship requiring disclosure. If no relationship exists, my Current Employer is not listed.

G. Strippoli reports the following:
Employer: University of Bari; Research Funding: Fresenius Medical Care - Research funding to University of Bari, Italy; Advisory or Leadership Role: Fresenius Medical Care; and Speakers Bureau: Fresenius Medical Care.

I understand that the information above will be published within the journal article, if accepted, and that failure to comply and/or to accurately and completely report the potential financial conflicts of interest could lead to the following: 1) Prior to publication, article rejection, or 2) Post-publication, sanctions ranging from, but not limited to, issuing a correction, reporting the inaccurate information to the authors' institution, banning authors from submitting work to ASN journals for varying lengths of time, and/or retraction of the published work.

Name: Giovanni Strippoli
Manuscript ID: CJASN-2025-000425
Manuscript Title: Baseline characteristics of populations included in randomized controlled trials of hemodiafiltration versus registry real-life populations: a systematic review
Date of Completion: July 7, 2025
Disclosure Updated Date: July 7, 2025

ASN Journal Disclosure Form

As per ASN journal policy, I have disclosed any financial relationships or commitments I have held in the past 36 months as included below. I have listed my Current Employer below to indicate there is a relationship requiring disclosure. If no relationship exists, my Current Employer is not listed.

M. Woodward reports the following:
Employer: The George Institute for Global Health; and Ownership Interest: MIS Statistical Consultants LLC.

I understand that the information above will be published within the journal article, if accepted, and that failure to comply and/or to accurately and completely report the potential financial conflicts of interest could lead to the following: 1) Prior to publication, article rejection, or 2) Post-publication, sanctions ranging from, but not limited to, issuing a correction, reporting the inaccurate information to the authors' institution, banning authors from submitting work to ASN journals for varying lengths of time, and/or retraction of the published work.

Name: Mark Woodward
Manuscript ID: CJASN-2025-000425
Manuscript Title: Baseline characteristics of populations included in randomized controlled trials of hemodiafiltration versus registry real-life populations: a systematic review
Date of Completion: June 18, 2025
Disclosure Updated Date: June 18, 2025

ASN American Society of Nephrology | JASN Journal of the American Society of Nephrology | KIDNEY360 Accessing Our World From Every Angle | CJASN Clinical Journal of the American Society of Nephrology

ASN Journal Disclosure Form

As per ASN journal policy, I have disclosed any financial relationships or commitments I have held in the past 36 months as included below. I have listed my Current Employer below to indicate there is a relationship requiring disclosure. If no relationship exists, my Current Employer is not listed.

T. Yuen reports the following:
Employer: Fresenius Medical Care

I understand that the information above will be published within the journal article, if accepted, and that failure to comply and/or to accurately and completely report the potential financial conflicts of interest could lead to the following: 1) Prior to publication, article rejection, or 2) Post-publication, sanctions ranging from, but not limited to, issuing a correction, reporting the inaccurate information to the authors' institution, banning authors from submitting work to ASN journals for varying lengths of time, and/or retraction of the published work.

Name: Tom Yuen
Manuscript ID: CJASN-2025-000425
Manuscript Title: Baseline characteristics of populations included in randomized controlled trials of hemodiafiltration versus registry real-life populations: a systematic review
Date of Completion: July 8, 2025
Disclosure Updated Date: July 8, 2025
