# Supplementary material for: Characteristics of Populations Included in Randomized Controlled Trials of Hemodiafiltration and Registry Real-Life Populations: A Systematic Review
Source: Clin J Am Soc Nephrol. 2025 Sep 12;21(1):95–107. doi: 10.2215/CJN.0000000855 (PMC13135059; doi:10.2215/CJN.0000000855)
Supplement: Supplementary file 2 [file cjasn-21-095-s002.pdf]

Supplementary Appendix

Supplementary Table 1. Search strategy for randomized controlled trials of hemodiafiltration versus hemodialysis.

Supplementary Table 2. Characteristics of the 2024 update of the Registries database curated by the Fresenius Quantitative Market Analysis team (data/search strategy available upon request).

Supplementary Table 3. Descriptive baseline characteristics of people enrolled in randomized controlled trials of hemodiafiltration versus hemodialysis.

Supplementary Table 4. Comparative analysis between estimates of patient baseline characteristics in randomized controlled trials – sorted by mean convection volume from lowest to highest.

Supplementary Table 5. Descriptive baseline characteristics of people from major kidney registries annual reports.

Supplementary Table 1

Search strategy for randomized controlled trials of hemodiafiltration versus hemodialysis.

Database	Search terms used
CENTRAL	1. MeSH descriptor Renal Replacement Therapy, this term only<br>2. MeSH descriptor Hemofiltration explode all trees<br>3. hemofiltrat* or haemofiltrat*:ti,ab,kw in Clinical Trials<br>4. hemodiafiltrat* or haemodiafiltrat*:ti,ab,kw in Clinical Trials<br>5. ultrafiltrat*:ti,ab,kw in Clinical Trials<br>6. biofiltrat*:ti,ab,kw in Clinical Trials<br>7. (acetate-free near/3 biofiltration):ti,ab,kw in Clinical Trials<br>8. (HDF or HF or AFB or RRT):ti,ab,kw in Clinical Trials<br>9. (#1 OR #2 OR #3 OR #4 OR #5 OR #6 OR #7 OR #8)<br>10. MeSH descriptor Renal Replacement Therapy, this term only<br>11. MeSH descriptor Renal Dialysis, this term only<br>12. MeSH descriptor Hemodialysis, Home, this term only<br>13. MeSH descriptor Kidney Failure, Chronic, this term only<br>14. (hemodialysis or haemodialysis):ti,ab,kw in Clinical Trials<br>15. (end-stage NEXT kidney):ti,ab,kw or (end-stage NEXT renal):ti,ab,kw or (endstage NEXT kidney):ti,ab,kw or (endstage NEXT renal):ti,ab,kw in Clinical Trials<br>16. (#10 OR #11 OR #12 OR #13 OR #14 OR #15)<br>17. (#9 AND #16)
MEDLINE	1. Renal Replacement Therapy/<br>2. Renal Dialysis/<br>3. Hemodialysis, Home/<br>4. Kidney Failure, Chronic/<br>5. (hemodialysis or haemodialysis).tw.<br>6. (end-stage kidney or end-stage renal or endstage kidney or endstage renal).tw.<br>7. (ESKD or ESKF or ESRD or ESRF).tw.<br>8. or/1-7<br>9. Renal Replacement Therapy/<br>10. exp Hemofiltration/<br>11. (hemofiltrat$ or haemofiltrat$).tw.<br>12. (hemodiafiltrat$ or haemodiafiltrat$).tw.<br>13. (acetate-free adj2 biofiltration).tw.<br>14. (HDF or HF or AFB or RRT).tw.<br>15. or/9-14
EMBASE	1. renal replacement therapy/<br>2. hemodialysis/<br>3. home dialysis/<br>4. (hemodialysis or haemodialysis).tw.<br>5. Chronic Kidney Disease/<br>6. Kidney Failure/<br>7. Chronic Kidney Failure/

	8. (end-stage renal or end-stage kidney or endstage renal or endstage kidney).tw.<br>9. (ESRF or ESKF or ESRD or ESKD).tw.<br>10. or/1-9<br>11. renal replacement therapy/<br>12. hemodiafiltration/<br>13. hemofiltration/<br>14. (acetate-free adj2 biofiltration).tw.<br>15. (HDF or AFB or HF).tw.<br>16. (extracorporeal adj RRT).tw.<br>17. (haemodiafiltrat$ or hemodiafiltrat$).tw.<br>18. (haemofiltrat$ or hemofiltrat$).tw.<br>19. or/11-18

Supplementary Table 2

Characteristics of the 2024 update of the Registries database curated by the Fresenius Quantitative Market Analysis team (data/search strategy available upon request).

- Database compiled through a structured methodology, beginning with a broad internet search using predefined keywords related to end-stage kidney disease, kidney registries, nephrology societies, transplantation, and dialysis statistics.
- Search strategy including Boolean operators and conducted using multiple search engines and indexed sources to maximize retrieval of relevant registries.
- Search supplemented with direct outreach to nephrologists, nephrology associations, and health agencies from various countries to verify and expand the registry list.
- Database subject to an annual review process where new entries are validated against official national health sources, nephrology societies, and government reports.
- “Ad hoc” updates throughout the year following the same systematic approach.
- Compiled registry list independently reviewed by nephrology experts to confirm accuracy and completeness.

Supplementary Table 3. Descriptive baseline characteristics of people enrolled in randomized controlled trials of hemodiafiltration versus hemodialysis.

Variable	Locatelli 1994	Wizemann 2000	Bolasco 2003	CONTRAST (Dutch) Study 2005	Schiffl 2007	ESHOL Study 2011	TURKISH HDF 2013	FRENCHIE 2017	FINESSE 2019	HDFIT 2019	CONVINCE 2023
No. of patients - N	380	44	146	714	76	906	782	381	124	195	1360
Race/ Ethnicity - n (%) [1]	Italy sites	Study in Germany, location of sites not specified	Italy sites	White: 300 (84.0)<br>Black: 58 (8.1)<br>Asian: 45 (6.3)<br>Other: 11 (1.5)	Germany sites	Spain sites (Catalonian area)	Turkey sites	France sites	White: 86 (69.4)<br>Black: NA<br>Asian: 9 (7.3)<br>Other: 29 (23.4)	White: 115 (59.0)<br>Others: 80 (41.0)	Western Europe: 441 (32.4)<br>Eastern Europe: 457 (33.6)<br>Southern Europe: 452 (33.2)
Convection volume in HDF group patients (L/ treatment or session) - mean ± SD [2]	Not available	Not available	Not available	20.7 ± 6.0	Not available	23.9 (23.6, 24.2) [5]	Not available	21.0 ± 5.3	24.7 (22.4-26.5) [6]	27.5 ± 2.9	25.2 (24.8, 25.7) [5]
Blood flow rate- Qb (ml/min) - mean ± SD [3]	Not available	400.0-500.0	≥300.0	300.5 ± 40.0	≥250.0	HD group: 380.0 (374.0, 387.0) [5]<br>HDF group: 392.0 (387.0, 398.0) [5]	294.0 ± 45.0	336.4 ± 41.9	302.0 ± 18.5	Not available	368.0 ± 55.0
UF volume (L/session) - mean ± SD	Not available	3.2 ± 1.0	2.8 (2.5 - 3.4) [6]	Not available	Not available	Not available	Not available	Not available	Not available	Not available	Not available
BMI postdialysis (kg/m^2) - mean ± SD	Not available	Not available	Not available	25.4 ± 4.8	Not available	Not available	24.8 ± 4.8	26.3 ± 4.9	Not available	26.7 ± 4.9	27.4 ± 5.6
Cardiovascular comorbidities - n (%) [4]	Not available	Coronary artery disease: 20 (45.5)<br>Cerebrovascular disease: 4 (9.1)<br>Valve replacement: 1 (2.3)<br>Amputation: 1 (2.3)	Hypertension: 83 (56.8)<br>Ischemic cardiopathy: 32 (21.9)<br>Peripheral arteriopathy: 14 (9.6)<br>Previous TIA: 17 (11.6)	Myocardial infarction: 98 (13.7)<br>PTCA or CABG: 116 (16.2)<br>Stroke or TIA: 109 (15.3)<br>Peripheral artery disease: 112 (15.7)<br>Amputation: 30 (4.2)	Not available	Not available	Not available	Hypertension: 284 (74.5)<br>Cardiopathy: 196 (51.4)<br>Arteriopathy: 160 (42.0)	Congestive heart failure: 13 (10.5)<br>Ischemic heart disease: 37 (29.8)<br>Cerebrovascular disease: 16 (12.9)<br>Peripheral vascular disease: 16 (12.9)	Congestive heart failure: 15 (7.7)<br>Coronary artery disease: 33 (16.9)	Coronary heart disease: 277 (20.4)
Dialysis time (minutes) - mean ± SD	Not available	273.1 ± 4.7	240.0 (210.0 - 240.0) [6]	226.5 ± 24.1	Not available	HD group: 234.1 (232.2, 236.1) [5]<br>HDF group: 235.8 (234.2, 237.3) [5]	Not available	235.8 ± 25.4	Not available	HD group: 235.0 (232.0 - 240.0) [6]<br>HDF group: 235.0 (233.0 - 240.0) [6]	HD group: 240.0 (240.0-245.0) [6]<br>HDF group: 240.0 (240.0-248.0) [6]

[1] Refer to figure 4 for the specific data on race & ethnicity from RCTs and Registries.

[2] Mean or median convection volume of HDF group patients during the study follow up period was collected from CONTRAST (Dutch) Study 2005, FINESSE 2019, and CONVINCE 2023. Mean convection volume of HDF group patients at follow up month 6 was collected from ESHOL Study 2011, FRENCHIE 2017, and HDFIT 2019.

[3] The blood flow rates collected from Bolasco 2003 and Schiffl 2007 are the study inclusion criteria. And that from Wizemann 2000 is the targeted dialysis method.

[4] Individual cardiovascular comorbidities data was not available from ESHOL Study 2011, but Charlson comorbidity index was reported - median (IQR): 7.0 (5.0 - 8.0)

[5] Only mean (95% CI) available

[6] Only median (IQR) available

Supplementary Table 4. Comparative analysis between estimates of patient baseline characteristics in randomized controlled trials – sorted by mean convection volume from lowest to highest.

**Characteristic** (mean ± SD or %)	**CONTRAST (Dutch)** 20.7 L	**FRENCHIE** 21.0 L	**ESHOL** 23.9 L	**FINESSE** 24.7 L	**CONVINCE** 25.2 L	**HDFIT** 27.5 L	**Difference by mean convection volume group (<23 L vs ≥23 L)** P value
Age	64.1 ± 13.7	76.2 ± 6.4	65.4 ± 14.4	63.5 ±14.7	62.4 ± 13.5	53.0 ± 15.1	0.10
Sex – female	37.7	39.9	33.1	44.4	36.0	28.7	0.32
Cardiovascular disease	43.8	66.4	NA	NA	45.0	22.1	0.19
Diabetes mellitus	23.8	38.6	24.9	35.5	35.0	34.9	0.86
Vascular access (arteriovenous fistula and graft)	93.4	NA	89.7	87.1	86.5	NA	< 0.001
Dialysis vintage	2.9 ± 2.9	4.8 ± 5.5	2.7 ± 2.9	3.4 ± 3.0	3.3 ± 3.5	NA	0.39

Supplementary Table 5. Descriptive baseline characteristics of people from major kidney registries annual reports.

Variable	United States Renal Data System (USRDS)	Japanese Society for Dialysis Therapy Renal Data Registry (JRDR)	European Renal Association (ERA) Registry	UK Renal Registry (UKRR)	Australia and New Zealand Dialysis and Transplant Registry (ANZDATA) - Australia	Australia and New Zealand Dialysis and Transplant Registry (ANZDATA)- New Zealand	Scottish Renal Registry (SRR)	Finnish Registry for Kidney Diseases (FIRR)
No. of patients - N	475142	316113	310686	25019	12861	2389	1937	1634
Race/Ethnicity - n (%)	White: 174,195 (36.6)<br>Asian: 243,53 (5.1)<br>Black: 161,865 (34.1)<br>Other: 113,625 (23.9)	Not available	Not available	White: 17,163 (68.6)<br>Asian: 3,827 (15.3)<br>Black: 3,102 (12.4)<br>Other: 900 (3.6)	Not available[6]	Not available[7]	Not available	Not available
Blood flow rate – Qb (ml/min) - mean ± SD	Not available	212.6 ± 37.5	Not available	Not available	Not available[3]	Not available[3]	Not available	Not available
Cardiovascular comorbidities - n (%)	Not available	Not available	Not available	Not available	CHD: 5719 (44.57)<br>CVD: 4355 (33.95)	CHD: 923 (38.72)<br>CVD: 646 (27.10%)	CHD: 494 (25.5%)<br>CVD: 799 (41.1)	CHD: 307 (20.2)<br>CVD: 438 (28.8)
Dialysis time (minutes) - mean ± SD	Not available	240-300[5] (HD: 72.7%, HDF: 77.9%)	Not available	240-300[1] (66.6%)	240-300[2] (90.8%)	240-300[4] (90.4%)	Not available	Not available

[1] Only categorical percentages of different session times are reported, M (SD) are not provided. <240: 33.1%, >300: 0.3%

[2] Only categorical percentages of different session times are reported, M (SD) are not provided. <240: 5.4%, >300: 3.9%

[3] Only categorical percentages of different blood pressure levels are reported, M (SD) are not provided.

[4] Only categorical percentages of different session times are reported, M (SD) are not provided. <240: 2.0%, >300: 7.6%

[5] Only categorical percentages of different session times are reported, M (SD) are not provided.

[6] Only data for 'Aboriginal and/or Torres Strait Islander': 2204 (14.2%) and 'Other': 13,007 (83.8%)

[7] Only data for 'Māori': 955 (30.3%) and 'Other': 2,177 (69.0%)
